# Academic dishonesty among university students: The roles of the psychopathy, motivation, and self-efficacy

**DOI:** 10.1371/journal.pone.0238141

**Published:** 2020-08-31

**Authors:** Lidia Baran, Peter K. Jonason

**Affiliations:** 1 Institute of Psychology, University of Silesia in Katowice, Katowice, Poland; 2 Department of General Psychology, University of Padua, Padua, Italy; 3 Institute of Psychology, Cardinal Stefan Wyszyński University in Warsaw, Warsaw, Poland; University of Lleida, SPAIN

## Abstract

Academic dishonesty is a common problem at universities around the world, leading to undesirable consequences for both students and the education system. To effectively address this problem, it is necessary to identify specific predispositions that promote cheating. In Polish undergraduate students (*N* = 390), we examined the role of psychopathy, achievement goals, and self-efficacy as predictors of academic dishonesty. We found that the disinhibition aspect of psychopathy and mastery-goal orientation predicted the frequency of students’ academic dishonesty and mastery-goal orientation mediated the relationship between the disinhibition and meanness aspects of psychopathy and dishonesty. Furthermore, general self-efficacy moderated the indirect effect of disinhibition on academic dishonesty through mastery-goal orientation. The practical implications of the study include the identification of risk factors and potential mechanisms leading to students’ dishonest behavior that can be used to plan personalized interventions to prevent or deal with academic dishonesty.

## Introduction

Academic dishonesty refers to behaviors aimed at giving or receiving information from others, using unauthorized materials, and circumventing the sanctioned assessment process in an academic context [1]. The frequency of academic dishonesty reported in research indicates the global nature of this phenomenon. For example, in a study by Ternes, Babin, Woodworth, and Stephens [2] 57.3% of post-secondary students in Canada allowed another student to copy their work. Similarly, 61% of undergraduate students in Sweden copied material for coursework from a book or other publication without acknowledging the source [3]. Working together on an assignment when it should be completed as an individual was reported by 53% of students from four different Australian universities [4], and copying from someone’s paper in exams at least once was done by 36% of students from four German universities [5]. Research shows that academic dishonesty is also a major problem at Polish universities. In the study by Lupton, Chapman, and Weiss [6] 59% of the students admitted to cheating in the current class, and 83.7% to cheating at some point during college. According to a report on the plagiarism in Poland, prepared by IPPHEAE Project Consortium, 31% of students reported plagiarizing accidentally or deliberately during their studies [7].

Existing academic dishonesty prevention systems include using punishments and supervision [8], informing students about differences between honest and dishonest academic actions [9], adopting university honor codes [10], and educating students on how to write papers and conduct research correctly [11]. Although these methods lead to a reduction of academic dishonesty (see [12]), their problematic aspects include the possibility of achieving only a temporary change in behavior, limited impact on students' attitudes towards cheating, and a long implementation period [13, 14]. Possible reasons for these difficulties include the fact that conventional prevention methods rarely address differences in students’ personality and academic motivations, which may be associated with a tendency to cheat. For example, previous studies have reported that negative emotionality was associated with positive attitudes toward plagiarism [15]; intrinsic motivation was associated with lower self-reported cheating [16]; and socially orientated human values were negatively, while personally focused values were positively correlated with academic dishonesty [17].

It is also important to remember that implementing the aforementioned methods of prevention will not lead to a reduction in academic dishonesty if faculty members do not follow and apply the established rules [18]. Faculty members often prefer not to take formal actions against dishonest students [19], and in many cases do not use the methods available to them to detect and prevent cheating [20]. However, when they do respond to academic dishonesty it is often in inconsistent ways [21]. This might suggest that, while dealing with students’ dishonesty, faculty members prefer to choose their own punitive and preventative methods, which may differ depending on the particular student and professor. If that is the case, then examining the role of individual differences in academic dishonesty could be useful not only to better understand the nature of academic transgressions but also to address faculty's informal ways of dealing with students' cheating.

The aim of the current study was to investigate relationships between personality, motivation, and academic dishonesty to understand the likelihood of cheating in academia more effectively and potentially inform faculty's personalized interventions. Of all the personality traits under investigation, psychopathy appears to be useful for this purpose, because it includes a tendency to be impulsive, to engage in sensation-seeking, and resistance to stress, all of which are associated with academic dishonesty [2]. Indeed, psychopathy is the strongest—albeit moderate in size (*r =* .27)—predictor of academic dishonesty according to a recent meta-analysis of 89 effects and 50 studies [22]. In the present study, we wanted to further examine the relationship between academic dishonesty and psychopathy by using the triarchic model of psychopathy distinguishing its three phenotypic facets: boldness, meanness, and disinhibition [23] which may reveal added nuance to how this personality trait relates to academic dishonesty.

Within the triarchic conceptualization of psychopathy, boldness represents self-assurance, fearlessness, and a high tolerance for stress and unfamiliarity; meanness captures interpersonal deficits such as lack of empathy, callousness and exploitativeness; and disinhibition represents the tendency towards impulsivity, poor self-regulation and focus on immediate gratification. Because of the different neurobiological mechanisms leading to the shaping of those aspects [24], it seems likely that the tendency towards academic dishonesty may have a different etiology depending on their levels. For students with high disinhibition, cheating may result from low self-control; for those with high meanness from rebelliousness with propensity to use others; and for bold ones from emotional resiliency and sensation-seeking [25–27]. However, because boldness constitutes fearlessness without failed socialization [28], breaking academic rules might not be the preferred way to look for excitement among bold students. Thus, our first goal was to examine the predictive power of boldness, meanness, and disinhibition in academic dishonesty.

Furthermore, we were interested if the relationships between the psychopathy facets and academic dishonesty would be mediated by individual differences in motivations for mastery and performance. Mastery motivation is fostered by the need for achievement and associated with learning to acquire knowledge, whereas performance motivation is geared towards reducing anxiety and related to learning to prove oneself to others [29]. We expect mediation for several reasons. First, undertaking actions motivated by achievement goals is predicted by the level of positive and negative emotionality and also by activity of the behavioral activation and inhibition system [30], which also correlate with the dimensions of the triarchic model of psychopathy [31]. Second, unrestrained achievement motivation partially mediates the relationship between psychopathy and academic dishonesty, suggesting a role of achievement in understanding the relationship between psychopathy and individual differences in the propensity to cheat [32]. Third, meanness and disinhibition are negatively and boldness positively correlated with conscientiousness and its facets [33, 34]. This fact may play an important role in students’ willingness to exert and control themselves to achieve academic goals and the particular way to do it [35]. Moreover, research on mastery-goal orientation suggests it is correlated negatively with academic dishonesty and views of the acceptability of academic dishonesty [36–38] and that the change from mastery to performance-based learning environment lead to increased levels of dishonesty [39].

Therefore, we hypothesized that students with a high level of disinhibition may have difficulties studying because of their need for immediate gratification and lack of impulse control, and in turn, cheat to pass classes. Bold students could want to acquire vast knowledge and high competences because of their high self-assurance, social dominance, and a high tolerance for stress without resorting to fraud. Lastly, students with a high level of meanness may be less prone towards mastery through hard work and learning because of their susceptibility to boredom, tendency to break the rules, and to exploit others to their advantage, perhaps by copying or using other students’ work. Because performance-goal orientation can be driven by the fear of performing worse than others, no specific hypothesis was generated regarding its relation to psychopathy (characterized by a lack of fear).

Besides behavioral tendencies based on personality traits and specific motives to learn, another closely related predictor of academic dishonesty is general self-efficacy. People with high levels of general self-efficacy exercise control over challenging demands and their behavior [40] and perform better in academic context because of their heightened ability to solve problems and process information [41]. On the other hand, low levels of general self-efficacy in the academic context can lead to reduced effort and attention focused on the task, which may result in a higher probability of frauds to achieve or maintain a certain level of academic performance [42, 43]. Because competence expectancies are important antecedents of holding an achievement goal orientation [44, 45] it seems possible that general self-efficacy might moderate the relation between psychopathy facets and academic dishonesty mediated by achievement goals. Thus, we hypothesize that high general self-efficacy will reduce the indirect effects for disinhibition and meanness (i.e., negative moderation effect) and amplify it for boldness (i.e., positive moderation effect).

In sum, we examine the relationships between three facets of psychopathy and academic dishonesty, the possible role of achievement goals as a mediators for those relations, and lastly the possible role of general self-efficacy as a moderator of those mediation models. By analyzing the facets of psychopathy independently, we can determine their unique relationship with the tendency to cheat and thus more accurately predict the risk of dishonest behavior for students with a high level of each of the facet. In addition, investigating indirect effects and interactions between personality and motivation may describe the psychological processes that may lead to cheating and can potentially be used in planning preventive actions.

## Materials and methods

### Participants and procedure

The participants were 390 Polish university students and residents (100% White, 74% female) with an average age of 23 (*SD* = 3.39, *Range* = 19–56) years. Participants self-identified as students in social sciences (17%), humanities (12%), science and technology (24%), law and administration (22%), and medical sciences (23%); 7 failed to respond (2%). In addition, participants were first-year (19%), second-year (16%), third-year (31%), fourth-year (13%), fifth-year (13%), and doctoral students (2%); 23 failed to respond (6%).

We established the required sample size as 290 participants, following Tabachnick and Fidell [46] guidelines and gave ourselves three months to collect it to avoid concerns with power and *p-*hacking, respectively. The study was approved by Ethics Committee of the Faculty of Pedagogy and Psychology (University of Silesia in Katowice) and was conducted online through the Webankieta platform to maximize the anonymity and security of the participants. An invitation to participate in the project was sent to 28 largest Polish universities by enrollment, with a request to publish it on the universities' websites. The link to the survey directed the participants to a detailed description of the research and the rules of participation. After consenting to participate, students completed online questionnaires and, at the end, they were asked if they wanted to receive a summary of the general results and take part in a prize drawing (after the end of the study, five randomly chosen participants received vouchers for online personal development courses). The present study was part of a larger investigation that aimed to examine psychological determinants and predictors of academic dishonesty.

### Measures

Psychopathy was measured with the TriPM-41 [34], the shortened Polish adaptation of the Triarchic Psychopathy Measure [47]. Participants rated statements on a 4-point scale (0 = *completely false*; 1 = *somewhat false*; 2 = *somewhat true*; 3 = *completely true*). Items were summed to create indexes for three subscales: disinhibition (16 items, e.g., “I jump into things without thinking”; Cronbach’s α = .83), meanness (10 items, e.g., “I don't have much sympathy for people”; α = .92), and boldness (15 items, e.g., “I'm a born leader”; α = .88).

Achievement goals were measured with the Polish translation of the Achievement Goals Questionnaire-Revised [29]. Participants reported their agreement (1 = *strongly disagree*; 5 = *strongly agree*) with statements such as “My aim is to completely master the material presented in this class” (i.e., mastery-goal orientation, 6 items) or “My aim is to perform well relative to other students” (i.e., performance-goal orientation, 6 items). Items were summed to calculate mastery (α = .80) and performance (α = .87) goal orientation indexes.

The Polish translation of the New General Self-Efficacy Scale [48] was used to measure general self-efficacy (e.g., “Even when things are tough, I can perform quite well”). Participants were asked how much they agreed (1 = *strongly disagree*; 5 = *strongly agree*) with eight items, which were summed to create the general self-efficacy index (α = .89).

Academic dishonesty was estimated with the Academic Dishonesty Scale [49], which is a list of 16 academically dishonest behaviors (e.g., “Using crib notes during test or exam” or “Falsifying bibliography”). Participants rate the frequency (0 = *never*; 4 = *many times*) of committing each behavior during their years of studies. Items were summed to create the academic dishonesty index (α = .83).

### Data analysis

Descriptive statistics were calculated with JASP (v0.9.0.0), correlations with STATISTICA (v13.1), and regression, mediation, and moderated mediation with SPSS (v25). In the mediation analysis we used model 4 in macro PROCESS 2.16.3 (10,000 bootstrapped samples) and for the moderated mediations model 7 in macro PROCESS 2.16.3 (10,000 bootstrapped samples). Analyzes were carried out on the responses from 390 fully completed surveys. Because of mixed results in previous studies concerning psychopathy and academic dishonesty levels in men and women (see [50, 51]) we conducted analyses on the overall results and also separately in each sex. The database was uploaded to Open Science Framework and is available under the following address: https://osf.io/frq9v/

## Results

Descriptive statistics, sex differences tests (see Bottom Panel), and correlations (see Top Panel) for all measured variables are presented in Table 1. Academic dishonesty was positively correlated with meanness and disinhibition, and negatively correlated with mastery-goal orientation and general self-efficacy. Mastery-goal orientation was positively correlated with boldness and general self-efficacy, and negatively correlated with meanness and disinhibition. Performance-goal orientation was positively correlated with meanness. General self-efficacy was positively correlated with boldness and negatively correlated with meanness and disinhibition. We found only three cases where these correlations were moderated by participant’s sex. The correlation between performance and mastery-goal orientation was stronger (*z =* -1.85, *p* = .03) in men (*r* = .51, *p* < .01) than in women (*r* = .34, *p* < .01). The correlation between mastery-goal orientation and meanness was stronger (*z =* 2.00, *p* = .02) in men (*r* = -.28, *p* < .01) than in women (*r* = -.05, *ns*). And the correlation between disinhibition and academic dishonesty was stronger (*z =* 1.72, *p* = .04) in women (*r* = .39, *p* < .01) than in men (*r* = .20, *p* < .01). If we adjust for error inflation for multiple comparisons (*p* < .007) for these moderation tests, none of the Fisher’s *z* tests were significant. Therefore, we conclude the correlations were generally similar in the sexes. Men scored higher than women on meanness and disinhibition.

**Table 1:** Descriptive statistics, sex differences, and correlations for psychopathy facets, achievement goals, general self-efficacy, and academic dishonesty.

	1	2	3	4	5	6	7
1. Boldness	-						
2. Meanness	-.01	-					
3. Disinhibition	-.20**	.24**	-				
4. Mastery-goal orientation	.16**	-.17**	-.20**	-			
5. Performance-goal orientation	.02	.11*	-.03	.34**	-		
6. General self-efficacy	.61*	-.12*	-.24**	.24**	.06	-	
7. Academic dishonesty	-.02	.10*	.32**	-.36**	-.07	-.11*	-
Overall: *M* (SD)	24.72 (8.56)	6.84 (5.95)	10.69 (6.87)	22.29 (4.05)	19.56 (5.23)	30.45 (5.85)	13.12 (10.21)
Women: *M* (SD)	24.26 (8.66)	5.89 (5.36)	10.09 (6.46)	22.50 (3.89)	19.45 (5.13)	30.61 (5.62)	12.70 (9.03)
Men: *M* (SD)	26.07 (8.13)	9.57 (6.81)	12.45 (7.70)	21.69 (4.43)	19.88 (5.51)	29.96 (6.50)	14.32 (13.04)
*t*-test	-1.83	-5.50*	-3.00*	1.73	-0.72	0.96	-1.37
Hedges’*g*	-0.21	-0.65	-0.35	0.20	-0.08	0.11	-0.16

We report Hedges’ *g* for effect size to adjust for unequal group sizes. Its interpretation is the same as the more common Cohen’s *d*.

* *p* < .05

** *p* < .01

To test the contribution of personality and motivation variables in predicting academic dishonesty, we conducted a standard multiple regression where the model explained 23% of the variance in academic dishonesty [*F*(6, 383) = 18.60, *p* < .001]. The residuals for boldness (*β* = .12, *p* = .04), disinhibition (*β* = .27, *p* < .01), and a mastery-goal orientation (*β* = -.39, *p* < .01) were correlated with academic dishonesty. Additional regression analysis revealed that both mastery-goal orientation and disinhibition strengthened the association between boldness and academic dishonesty, which on its own was not a predictor of the frequency of cheating–suppressor effect (results of hierarchical regression showed that after adding boldness to the model explained variance increased by 1% [Δ*F*(1, 383) = 4.40, *p* = .04]).

To examine whether achievement goals mediated the associations between psychopathy and academic dishonesty we conducted a series of mediation analyses.

As shown in Table 2 (see Left Panel), mastery-goal orientation mediated the relation between facets of psychopathy and academic dishonesty (i.e., none of the indirect effects CIs contained zero), and performance-goal orientation was not a mediator of those relations (see Right Panel; all of the indirect effects CIs contained zero). Mastery-goal orientation mediated relation between disinhibition and academic dishonesty (i.e., initial 𝛽_Step 1_ = .32, *p* < .001; 𝛽_Step 2_ = .24, *p* < .001), and the relationship between meanness and academic dishonesty (i.e., 𝛽_Step 1_ = .10, *p* < .05; 𝛽_Step 2_ = .05, *p* = .29). Initial non-significant negative relation between boldness and academic dishonesty (𝛽 = -.0001, *p* = .99) stayed unrelated after adding mastery-goal orientation to the model, but the value for the relation coefficient was higher and positive (𝛽 = .07, *p* = .12) suggesting a nonsignificant suppression effect.

**Table 2:** Mediation analysis concerning whether achievement goals mediate the relation between psychopathy facets and academic dishonesty.

	Mastery-goal orientation				Performance-goal orientation			
	*ab*	*SE*	95% CI	*z*	*ab*	*SE*	95% CI	*z*
Boldness	-0.07	0.02	-0.12, -0.03	-3.35*	-0.001	0.01	-0.02, 0.01	-0.24
Meanness	0.05	0.02	0.01, 0.11	2.66*	-0.01	0.01	-0.03, 0.002	-1.17
Disinhibition	0.08	0.02	0.04, 0.13	4.03*	0.002	0.01	-0.003, 0.02	0.53

*ab* = coefficient for the indirect effect; 95%CI = 95% confidence intervals; *z* = Sobel’s test for indirect effect.

* *p* < .01

To test if the level of general self-efficacy moderated the aforementioned relationships between psychopathy, achievement goals, and academic dishonesty we ran a series of moderated mediations. Index for moderated mediation was significant only for the model with disinhibition and mastery-goal orientation (**-**0.03; 95% CI: -0.70, -0.003), however, the same analyses ran separately for men (**-**0.03; 95% CI: -0.13, 0.05) and women (**-**0.04; 95% CI: -0.08, -0.01) revealed moderated mediation only in women (therefore, we do not report these analyses in men; they can be obtained from the first author). Estimates for that model are presented in Table 3.

**Table 3:** Estimates for moderated mediation for the relation between disinhibition, mastery-goal orientation, general self-efficacy, and academic dishonesty in women.

		Mastery-goal orientation				Academic dishonesty		
		*B*	*SE*	95% CI		*B*	*SE*	95% CI
Mastery-goal orientation (mediator)		-	-	-	B	-0.31**	0.06	-0.41, -0.20
Disinhibition (predictor)	A1	-0.24**	0.06	-0.35, -0.12	C’	0.23**	0.06	0.12, 0.34
General self-efficacy (moderator)	A2	0.14*	0.06	0.02, 0.25		-	-	-
Interaction disinhibition × general self-efficacy	A3	0.12*	0.05	0.02, 0.23		-	-	-
***Model summary***		*R*^2^ = .09; *F*(3, 286) = 9.85**				*R*^2^ = .18; *F*(2, 287) = 31.08**		

*B* = regression coefficients; *SE* = standard error; 95% CI = 95% confidence intervals; A1, A2, A3, B, and C’ are the paths in the moderated mediation model.

* p < .01

** p < .001

Women with high levels of disinhibition manifesting low level of mastery-goal orientation (see Left Panel, line A1) declared higher levels of academic dishonesty (see Right Panel, line B). An interaction between disinhibition and general self-efficacy (see Left Panel, line A3) with the significant, negative index for moderated mediation means that the indirect effect of disinhibition on academic dishonesty through mastery-goal orientation is negatively moderated by general self-efficacy. The higher the level of the moderator, the weaker the effect of mediation, and for moderator values above one standard deviation from mean mediation become non-significant (95% CI: -0.01, 0.09). In sum, the mastery-goal orientation partially mediated the associations that disinhibition had with academic dishonesty, however, this effect was absent for people with high levels of general self-efficacy.

## Discussion and limitations

Psychopathy is an important predictor of engaging in unethical behaviors [52], including in an academic context [53]. In the present study, we examined the relationships between facets of psychopathy, as described in the triarchic model of psychopathy (i.e. disinhibition, meanness, and boldness), and the frequency of academic dishonesty among students. We revealed that students with higher levels of meanness and disinhibition, but not boldness, reported more frequent academic dishonesty during their tertiary study.

In the case of meanness, this relationship may indicate a tendency for dishonesty resulting from a lack of fear and, consequently, a diminished impact of the perceived risk of being caught cheating, sensation-seeking that involves engaging in destructive behavior regardless of possible negative consequences of such actions, and a propensity to exploit other student’s work or knowledge to pass classes [23, 54]. The association between disinhibition and academic dishonesty may indicate impulsive cheating resulting from self-control problems (see [55]), and an inability to predict possible negative consequences of cheating [26]. The fact that academic dishonesty and boldness were uncorrelated may indicate that even though bold students can perform successfully in stressful situations and have high levels of sensation-seeking, those features are unrelated to the tendency to cheat in the academic context. It confirms that the “successful psychopath” [56] may be characterized by boldness but not antisocial behavior. Of all the facets of psychopathy, disinhibition was the strongest predictor of academic dishonesty, which confirms the role of impulsivity in predicting risky behavior [57, 58], and the role of delaying gratification in refraining from academic transgressions [59].

Beyond these basic associations, we also examined the role of achievement goals as mediators for the relationships between psychopathy facets and academic dishonesty. Mastery-goal orientation mediated the relationships between two psychopathy facets and academic dishonesty. Both meanness and disinhibition led to low levels of students’ mastery-goal orientation which, in turn, contributed to cheating in the academic context. Low mastery-goal orientation might result from the fact that those who are characterized by meanness may have a propensity to be rebellious (e.g., disregard for formal responsibilities, low diligence, and sensitivity to rewards) and those who are characterized by disinhibition may have a propensity for impulsivity (e.g., inability to postpone gratification or control impulses, high behavioral activation system). Without motivation to acquire knowledge, students may cheat to achieve academic goals with no regard to the fairness (i.e., high meanness) or the consequences (i.e., high disinhibition) of their actions [31–33]. In the case of boldness, the result of the mediation analysis might indicate a cooperative or reciprocal suppression effect, however, it should not be trusted because the main effect path did not pass the null hypothesis threshold when the potential suppressor was included in the model. Nonetheless, it seems possible that a particular configuration of boldness and disinhibition could lead to the interactive effect of those facets on the other variables [26]. Performance-goal orientation did not mediate the relationships between psychopathy facets and academic dishonesty, probably because bold, mean, and disinhibited students are not motivated by the fear to perform worse than others [60].

Lastly, we tested if general self-efficacy acts as a moderator of these mediation models and found evidence that it moderated the indirect effect of disinhibition on academic dishonesty through mastery-goal orientation. This means that disinhibited students who have a high sense of perceived ability to control their chances for success or failure, might be able to overcome the tendency to cheat resulting from their personality (i.e., high impulsiveness), and motivational (i.e., low motivation to learn) predispositions. However, that effect was found only for women, limiting any insights that can be drawn about men. Previous research showed that an increase in general self-efficacy reduced the risk of suicide among women [61]. Moreover, Portnoy, Legee, Raine, Choy, and Rudo-Hutt [62] found that low resting heart rate was associated with more frequent academic dishonesty in female students, and that self-control and sensation-seeking mediated this relationship. Thus, along with the observed lower level of disinhibition for female students, it appears that self-regulation abilities may play a different role for men and women’s performance, and also that deficits in self-control might not lead to the same behavioral tendencies in the sexes (see [63]). However, because of the cross-sectional nature of our study and an uneven number of men and women in the sample, this needs to be investigated further.

In the present study, we aimed to combine personality and motivation variables to describe the possible process leading to academic dishonesty assessed with a behavioral measure. Because Polish students do not constitute a typical W.E.I.R.D. sample (i.e., Western, Educated, Industrialized, Rich, and Democratic), presented results can be used to generalize conclusions from research on academic dishonesty beyond typical W.E.I.R.D cultures. However, our study is not without limitations. First, the measurement of academic dishonesty was based on self-report, which, even after maximizing anonymity of the measurement, might have attenuated our results concerning the frequency of cheating. Thus, future studies should focus on measuring actual dishonest academic behavior. Second, we examined academic dishonesty as an overall frequency of committing different acts of cheating, which reflects the general propensity to cheat. It could be useful to further investigate the predictive power of described models in experiments, focused on the specific type of dishonest behavior. Third, the obtained range of academic dishonesty scores might result from sampling bias, which would require using different sampling procedure in future studies, or from non-normal distribution of academic dishonesty, which would be consistent with the results of the previous studies [2–4]. Fourth, we tested mediation models in a cross-sectional study with a one-time point measurement, which require cautious interpretation. Future studies could use longitudinal methods; starting at the beginning of the first year and continuing over the course of their studies to capture the influence of personality, achievement goals, and general self-efficacy on the academic dishonesty of students in a more robust manner. Despite these shortcomings, our study is the first attempt (we know of) to integrate the triarchic model of psychopathy, general self-efficacy, and achievement goals to predict academic dishonesty, showing potential for further investigation in this area.

### Implications and conclusions

Preventing academic dishonesty is often made difficult by the lack of centralized and formalized university policies concerning cheating, faculty reluctance to take formal action against dishonest students, and limited attention paid to students’ personal characteristics associated with a tendency to cheat [64]. Based on the results of our study, lecturers might overcome those difficulties by: maximizing the amount of oral examinations to deal with the risk of cheating by disinhibited and mean students; enhancing students’ mastery-goal orientation, for example, by increasing use of competency-based assessment; enhancing students’ self-efficacy in academic context, for example, by providing spaced assessed tasks, and the opportunity to practice skills needed for their fulfillment. In the case of dealing with actual dishonest behavior, the fact that teachers prefer to warn students rather than fail them [19] might suggest indifference to academic integrity rules, reluctance to initiate time-consuming formal procedures against cheating, or teachers’ preference toward autonomy to deal with dishonesty. Therefore, a useful solution could be to assess which areas need to be improved for a particular student (e.g., knowledge about plagiarism, ability to delay gratification, or treating acquisition of knowledge as a value) and to allow the teacher to choose an effective way to remedy them.

In sum, we presented evidence that disinhibition and meanness are associated with the frequency of committing academic dishonesty. We described the possible underlying mechanism of those relations involving mediation effects of the mastery-goal orientation and, in the case of disinhibition, also a moderation effect of the general self-efficacy. Our research can be used by teachers to better identify factors conducive to dishonesty and to modulate their responses to fraud based on the personality and motivational predispositions of students.

## Supporting information

S1 Table Descriptive statistics and correlations for academically dishonest behaviors. (DOCX)
