# Supplementary material for: Academic dishonesty among university students: The roles of the psychopathy, motivation, and self-efficacy
Source: PLoS One. 2020 Aug 31;15(8):e0238141. doi: 10.1371/journal.pone.0238141 (PMC7458306; doi:10.1371/journal.pone.0238141)
Supplement: S1 Table — (DOCX) [file pone.0238141.s001.docx]

| Dishonest behavior | *M* | *SD* | GSE | MGO | PGO | Boldness | Meanness | Disinhibition |
| --- | --- | --- | --- | --- | --- | --- | --- | --- |
| Using crib notes on a test. | 1.74 | 1.32 | .02 | -.29** | -0.02 | 0.05 | -0.05 | 0.08 |
| Copying from another student during a test. | 1.76 | 1.22 | -.08 | -.33** | -0.10 | -0.06 | -0.06 | .15** |
| Using unfair methods to learn what was on a test before it was given. | 4.43 | 1.32 | -.03 | -.16** | -0.06 | -0.001 | 0.06 | .15** |
| Copying from another student during a test without their knowledge. | 0.57 | 0.98 | -.19** | -.28** | -.11* | -.13* | 0.08 | .20** |
| Helping someone else to cheat on a test. | 2.00 | 1.19 | .01 | -.16** | -0.05 | 0.09 | -0.002 | .12* |
| Cheating on a test in any other way. | 0.77 | 1.19 | .02 | -.27** | 0.02 | 0.07 | 0.10 | .21** |
| Copying material and turning it in as your own work. | 0.29 | 0.83 | -.13* | -.17** | -0.01 | -.15** | .11* | .23** |
| Fabricating or falsifying a bibliography. | 0.26 | 0.71 | -.08 | -.10* | -0.05 | 0.03 | 0.10 | .20** |
| Turning in work done by someone else. | 0.20 | 0.67 | -.01 | -0.08 | 0.01 | -0.03 | 0.08 | .23** |
| Receiving substantial, unpermitted help on an assignment. | 0.39 | 0.89 | -.08 | -.18** | -0.07 | 0.01 | .11* | .20** |
| Collaborating on an assignment when the instructor asked for individual work. | 0.97 | 1.22 | -.10 | -.25** | -0.05 | -0.01 | .13** | .19** |
| In a course requiring computer work, copying another student’s materials rather than doing your own. | 0.35 | 0.78 | -.06 | -.17** | 0.03 | -0.01 | .13** | .17** |
| Browse Internet sources for ideas without giving the source. | 1.29 | 1.22 | -.11* | -.19** | 0.03 | -0.04 | .11* | .22** |
| Submit another’s material as your own – from another student, a book, or the Internet – without giving credit. | 0.25 | 0.74 | -.12* | -.19** | 0.03 | -.10* | .18** | .23** |
| Used a cell phone to text message for help during an exam. | 0.33 | 0.79 | -.02 | -.18** | -0.04 | 0.04 | -0.01 | .21** |
| Used a cell phone or another device to photograph an exam. | 0.53 | 0.94 | -.001 | -.22** | -0.06 | 0.03 | 0.06 | .23** |

**S1 Table. Descriptive statistics and correlations for academically dishonest behaviors.**

GSE = general self-efficacy, MGO = mastery-goal orientation, PGO = performance-goal orientation.

* *p* < .05. ** *p* < .01
